# Emerging Promise of Computational Techniques in Anti-Cancer Research: At a Glance

**DOI:** 10.3390/bioengineering9080335

**Published:** 2022-07-25

**Authors:** Md. Mominur Rahman, Md. Rezaul Islam, Firoza Rahman, Md. Saidur Rahaman, Md. Shajib Khan, Sayedul Abrar, Tanmay Kumar Ray, Mohammad Borhan Uddin, Most. Sumaiya Khatun Kali, Kamal Dua, Mohammad Amjad Kamal, Dinesh Kumar Chellappan

**Affiliations:** 1 Department of Pharmacy, Faculty of Allied Health Sciences, Daffodil International University, Dhaka 1207, Bangladesh; mominur.ph@gmail.com (M.M.R.); md.rezaulislam100ds@gmail.com (M.R.I.); firoza29-1292@diu.edu.bd (F.R.); saidur29-1299@diu.edu.bd (M.S.R.); shajib.ph@diu.edu.bd (M.S.K.); sayedul29-1397@diu.edu.bd (S.A.); tanmayray.pharma@gmail.com (T.K.R.); borhan29-1276@diu.edu.bd (M.B.U.); mail.mskk@gmail.com (M.S.K.K.); prof.ma.kamal@gmail.com (M.A.K.); 2 Discipline of Pharmacy, Graduate School of Health, University of Technology Sydney, Sydney, NSW 2007, Australia; Kamal.Dua@uts.edu.au; 3 Faculty of Health, Australian Research Centre in Complementary and Integrative Medicine, University of Technology Sydney, Ultimo, NSW 2007, Australia; 4 Uttaranchal Institute of Pharmaceutical Sciences, Uttaranchal University, Dehradun 248007, India; 5 Institutes for Systems Genetics, Frontiers Science Center for Disease-Related Molecular Network, West China Hospital, Sichuan University, Chengdu 610041, China; 6 King Fahd Medical Research Center, King Abdulaziz University, Jeddah 21589, Saudi Arabia; 7 Enzymoics, 7 Peterlee Place, Novel Global Community Educational Foundation, Hebersham, NSW 2770, Australia; 8 Department of Life Sciences, School of Pharmacy, International Medical University, Bukit Jalil, Kuala Lumpur 57000, Malaysia

**Keywords:** cancer, immune system, computational tools, computer-based drug design, machine learning algorithms

## Abstract

Research on the immune system and cancer has led to the development of new medicines that enable the former to attack cancer cells. Drugs that specifically target and destroy cancer cells are on the horizon; there are also drugs that use specific signals to stop cancer cells multiplying. Machine learning algorithms can significantly support and increase the rate of research on complicated diseases to help find new remedies. One area of medical study that could greatly benefit from machine learning algorithms is the exploration of cancer genomes and the discovery of the best treatment protocols for different subtypes of the disease. However, developing a new drug is time-consuming, complicated, dangerous, and costly. Traditional drug production can take up to 15 years, costing over USD 1 billion. Therefore, computer-aided drug design (CADD) has emerged as a powerful and promising technology to develop quicker, cheaper, and more efficient designs. Many new technologies and methods have been introduced to enhance drug development productivity and analytical methodologies, and they have become a crucial part of many drug discovery programs; many scanning programs, for example, use ligand screening and structural virtual screening techniques from hit detection to optimization. In this review, we examined various types of computational methods focusing on anticancer drugs. Machine-based learning in basic and translational cancer research that could reach new levels of personalized medicine marked by speedy and advanced data analysis is still beyond reach. Ending cancer as we know it means ensuring that every patient has access to safe and effective therapies. Recent developments in computational drug discovery technologies have had a large and remarkable impact on the design of anticancer drugs and have also yielded useful insights into the field of cancer therapy. With an emphasis on anticancer medications, we covered the various components of computer-aided drug development in this paper. Transcriptomics, toxicogenomics, functional genomics, and biological networks are only a few examples of the bioinformatics techniques used to forecast anticancer medications and treatment combinations based on multi-omics data. We believe that a general review of the databases that are now available and the computational techniques used today will be beneficial for the creation of new cancer treatment approaches.

## 1. Introduction

Cancer is a group of diseases marked by uncontrolled cell division which spreads to or invades other parts of the body [1,2,3,4,5,6,7]. It is a devastating disease that affects both the old and the young. In 2012 alone, there were an estimated 14.1 million new cases and 8.2 million deaths worldwide. It has been predicted that 22 million new cancer cases and 13 million deaths may occur in the next two decades [8]. The inherent complexity and heterogeneity of cancer have proven to be a substantial hurdle for finding effective therapies, which are typically exacerbated by tumor proliferation and metastasis [9]. Based on the affected cell types, cancer can be classified into over 200 classes. Detailed information on these classes can be obtained from The National Cancer Institute, e.g., their origins, relevant therapies, and Food and Drug Administration (FDA)-authorized medications, among other things [10]. According to the most recent 2015 Cancer Trends Progress Report, death rates due to cancer have dropped since the early 1990s [11]. The route to discovering novel drugs has always been a winding one. The main goal of drug development is to find a single compound or a combination of compounds that can deliver the required medical effects. Thus, research in bioinformatics, cellular and molecular biology, experimental medicine, genetics, genomics, medicinal chemistry, pharmacology, pharmacokinetics and metabolism, proteomics, structural biology, and tumor biology is critical for developing an effective anticancer drug [12,13,14,15].

Drug discovery relies heavily on pre-clinical screening of promising molecules [16,17]. Animal trials and in vitro drug screening have improved the methods of compound selection with impressive results [18,19,20,21,22]. In cancer treatment discovery, however, the investigational assays that are employed for analyzing small compounds are often costly [23,24,25] and time-consuming. As a result, more effective techniques to develop traditional drugs are needed. Discovering new uses for existing medications is far more cost-effective than searching for new cancer-fighting therapies. It is possible to anticipate anticancer therapeutic efficacy based on drug repositioning using generated multi-omics data. Dealing with patient heterogeneity is a major difficulty in contemporary cancer therapy. It has been known for over 50 years that various types of cancer patients will respond very differently when given the same treatment. Combination therapy, which employs a number of different medications in order to obtain better clinical results, is also commonly used to treat cancer. Combination therapies/medicines have numerous advantages over monotherapy [26,27,28,29].

We examine how these approaches have been successfully employed in assisted separation. Problem patterns in the handling of drug design for cancer treatments, establishing research gains that are integrated with in silico drugs, have the potential to yield ingenious anticancer medications.

## 2. Anticancer Drug Target Prediction

Only about 400 encoded proteins have been proven to be helpful in developing medicines [30]. In contrast, there is a slew of potential therapeutic atomic targets for treating cancer [31]. The conventional approach is often based on the “one molecule, one target, one disease” paradigm, which avoids drug–protein interactions. However, it is worth mentioning that many challenging disorders are associated with a variety of objective proteins [32,33,34]. Furthermore, due to certain medicines having “poly-pharmacological” characteristics, unanticipated drug functionalities resulting from off-targets that are uncontrolled and unavoidable can lead to unpleasant side effects [7,35,36,37,38,39]. Sildenafil (Viagra), for example, is currently used to treat erectile dysfunction in men but was primarily designed for the treatment of angina [40]. Several medicines, including anticancer drugs, are yet to be discovered, as their associated target proteins (both primary and non-target) are unknown [7,41,42,43,44,45,46,47]. Furthermore, several therapeutic cancer targets remain “unchecked” by pharmaceutical establishments. Phosphatases, transcription factors, and representatives of RAS are categorized as “undruggable” because they require active enzymatic sites [48]. The explanation for all possible novel ligand-bound sections has been highlighted as a significant step in changing a medication and repurposing it, to establish the ability of modern medicines to deal with new symptoms. As a result, new long-conditioned bioinformatics target prediction is fundamental for identifying a reliable drug target indicator. Several new web servers have been developed based on drug–target interactions, each with its own set of databases and reliable prediction tools. Moreover, computational drug design approaches are used to investigate provable protein–drug interactions. Net-based models, and motor-review-based designs specifically, have emerged as powerful tools. The Genetic Association Database is a repository for research on complicated diseases and disorders and human genetic associations. This database’s objective is to track the user to find polymorphisms that are medically significant. GAD offers a comprehensive, open, web-based collection of molecular, clinical, and study characteristics for more than 5000 human genetic association studies. With the aid of contemporary high-throughput test techniques and up-to-date annotated molecular nomenclature, this database enables the systematic examination of complicated common human genetic diseases. All the datasets can be obtained from the website (Table 1 and Table 2) [29].

## 3. Computer-Aided Drug Discovery and Design

Max Perutz and John Kendrew were awarded the Nobel Prize in Chemistry in 1962 (myoglobin) for introducing the early hearing long-resolution protein system. They had received grants to collaborate with others in the crystallographic determination of protein structure, most recently with Brian Kobilka and Robert Lefkowitz for research on the constituents of G-protein-coupled receptors (GPCRs) [49]. The interest in searching for potential molecules that may be developed into effective formulations developed naturally, with the emphasis on the chemical orientation and the three-dimensional relative position of each atom in a target moiety, from an insensitive screening procedure that relied on the chance to uncover molecular hits to a process established as “normal” medicine discovery and design. Capoten (captopril), the first-ever drug that acted by inhibiting angiotensin-converting enzyme (ACE), was the first medication that benefited from the improvement in utilizing structural information, in the 1980s. In 1997, the HIV protease inhibitor nelfinavir mesylate (Viracept) became the first medication to be authorized in the United States entirely as a result of the structure of the active target site. These breakthroughs were just the beginning of the hunt for better, quicker, and less costly methods, in addition to computational methods and strategies for designing new medicines or improving existing ones. Efforts have also been made to analyze more compounds vis-a-vis the target (the screening method) in less time and to gather essential knowledge and experience to establish a library of molecules of a variety of chemicals for explicit future screening (Table 3). Along with the fragment’s 3D structure, other factors taken into account include the atoms’ van der Waals radii, electrostatic charges, dipoles, flourishes, and dihedral angles. Scientists can now utilize virtual or in silico studies to model real-world processes using computing resources such as sophisticated agencies or supercomputers. This development has paved the way for discovering more efficient and selective medicines with fewer side effects in a cost-efficient and less time-consuming manner.

With the use of these technologies, it is now feasible to test more chemicals in a shorter time with lower cost (virtual screening). Computer-assisted drug discovery and design (CADDD) has led to scientists making in silico breakthroughs. Computer models of synthetic techniques have shown some promise. These advancements may be seen in the resolution of 3D structures using computer models, the optimization and creation of new compounds, and the investigation and characterization of the atomic processes of earlier medications or natural items. In addition, they expand the definition of orthosteric medication to include allosteric modulators and biopic medicines in the quest for therapeutic molecules. Orthosteric medications are those that bind to a target at a particular active site.

Arieh Warshel, Michael Levitt, and Martin Karplus received the 2013 Nobel Prize in Chemistry for their contributions to the modeling of complex systems in chemical and biological processes and interactions from a computational and physical perspective.

Many systems are now being developed to boost and support the drug discovery process. CADDD has greatly improved since its initiation, with significant contributions from many other research organizations worldwide. It is now possible to incorporate multiple data sources to speed up the discovery of novel medicines that reshape the behavior of therapeutically important protein targets and enhance early-stage pharmaceutical research. Advances in CADDD automation have resulted in an environment that supports the evaluation and synthesis of huge numbers of compounds in less time, making the drug discovery process faster and more cost-effective. CADDD is now a broadly accepted term for describing the computational tools and also a method of storing, managing, analyzing, and modeling compounds that are useful at every step of a drug development project, from lead compound identification to intensification, target description, and validation, even in pre-clinical studies [50,51,52,53,54].

### 3.1. Computer-Aided Drug Design Based on Ligands

The ligand-based computer-aided drug design (LB-CADD) method investigates ligands that are known to interact with targets of interest. These methods make use of a collection of reference structures acquired from substances that are known to interact effectively and assesses their 2D or 3D structures. The overall goal is to modify these mixtures in a way that preserves the physicochemical characteristics most critical to their desired interactions while removing extra information that is irrelevant to those interactions. Since this does not necessitate understanding the structure of interest, it is perceived as an indirect approach to dealing with medication discovery. The building of a QSAR demonstration that predicts biologic movement from synthetic structures, or the determination of mixtures based on the similarity of a substance to recognized active molecules using similarity measures are the two key techniques of LB-CADD. The difference between the two techniques is that the latter gives more weight to the compound’s structure and highlights how this affects the flow of biological information more than the former. The techniques are related to in silico screening for novel compounds with intriguing biologic actions, quick drug development, hit-to-lead assays, and improvement of DMPK/ADMET characteristics. The foundation of LB-CADD is the idea that atoms that are fundamentally analogous are likely to exhibit comparable properties. LB-CADD methods rather than SB-CADD procedures can also be used when the biologic target’s structure is unknown. Additionally, ligand-based virtual high-throughput screening (LB-vHTS) techniques frequently identify dynamic mixtures that are more potent than those identified by SB–vHTS [55,56,57].

### 3.2. Drug Design Using Structure-Based Computer Assistance

The three-dimensional structure is used in structure-based computer-assisted drug design to find irritants that are rationally tied to their coupling pocket and may subsequently become excellent drug candidates. Either atomic magnetic resonance (NMR), homology modeling, or X-ray protein crystallography may be used to determine the three-dimensional structure. The final strategy makes use of a homologous protein whose structure has been determined by one of the earlier procedures. Receptor ligand docking and rescoring methods are the core building blocks of calculations in the field of structure-based medicine configuration. The goal of receptor ligand docking is to predict the binding of a ligand in the coupling pocket of a receptor using just the topology (or information adaptation) of the former and the 3D directions of the latter. In this way, docking systems often include a calculation that generates a wide range of postures to be scored by the scoring capacity and a scoring capacity that measures the collaborative liveliness of each (moderate) posture. Most of the time, scoring abilities can be separated into learning-based and observation-based categories. The latter employ a variety of (frequently physically inspired) terms whose coefficients are advanced using a specific informational index with known binding free energies, in contrast to the former, which use a reversal of the Boltzmann factor to compute scores from the repetition of various perceptions [55,58,59].

**Table 3:** Selected inhibitors developed using computational chemistry and rational drug design strategies [53].

Compound	Function	Therapeutic Area	Approval Time	References
Captopril	ACE inhibitor	Diabetic nephropathy, hypertension, congestive heart failure, myocardial infarction	1975	[60,61]
Cimetidine	H_2_ receptor antagonist	Heartburn and peptic ulcer therapy	1978	[62]
Dorzolamide	Inhibitor of carbonic anhydrase	Antiglaucoma agent	1989	[63,64]
Saquinavir	Inhibitor of HIV-1 protease	Antiretroviral medication to treat HIV or AIDS	1995	[65,66]
Zanamivir	Inhibitor of neuraminidase	Antiviral (influenza A and influenza B)	1999	[67,68,69,70]
Nelfinavir	Inhibitor of HIV protease	Antiretroviral medication to treat HIV or AIDS	1999	[71]
Lopinavir	HIV protease inhibitor with peptidomimetic properties	Antiretroviral medication used to treat HIV/AIDS in patients who have developed resistance to other protease inhibitors.	2000	[72]
Darunavir	Inhibitor of nonpeptic HIV protease	Antiretroviral for HIV/AIDS	2006	[73,74]
Imatinib	Inhibitor of tyrosine kinase	Chronic myeloid leukemia	1990	[75,76]
Gefitinib	Epidermal growth factor receptor (EGFR) kinase inhibitor	Non-small-cell lung cancer (NSCLC)	2003	[77,78]
Erlotinib	EGFR kinase inhibitor	Pancreatic cancer, NSCLC	2005	[79]
Sorafenib	Vascular endothelial growth factor receptor (VEGFR) kinase inhibitor	Thyroid cancer, renal cancer, liver cancer	2005	[80,81]
Lapatinib	Erb-B2 receptor tyrosine kinase 2 (ERBB2)/EGFR inhibitor	Breast cancer	2007	[82,83]
Abiraterone	Inhibitor of androgen synthesis	Hormone refractory prostate cancer or metastatic castration-resistant prostate cancer	2011	[84,85]
Crizotinib	Anaplastic lymphoma kinase (ALK) inhibitor	NSCLC	2011	[86,87,88]

## 4. Anticancer Small Organic Molecules Design via a Computational Approach

**Figure 1:**
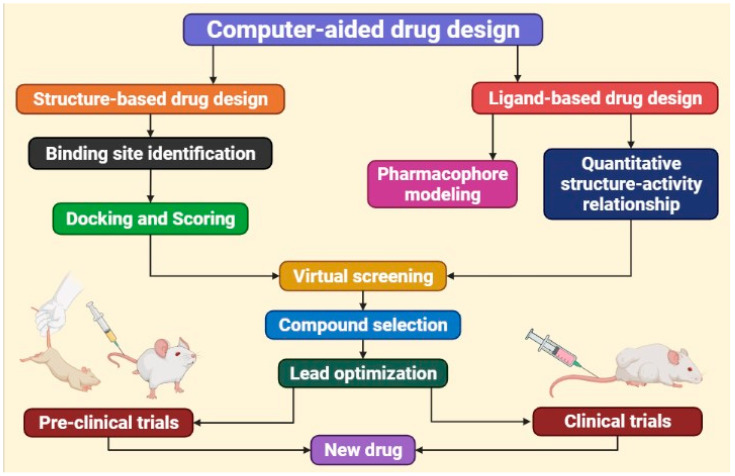
New drug development process using computer-aided drug design approach.

Conventionally, there are two different approaches to computer-aided design of anticancer small organic compounds: ligand-based drug design (LBDD) and structure-based drug design (SBDD). When a large number of particles can contain a receptor, LBDD is preferred [54]. SBDD, on the other hand, necessitates familiarity with the recipient′s three-dimensional structure. In most cases, the target structure is obtained using special experimental techniques such as X-ray, NMR, or Cryo-EM techniques; however, when the target structure is missing homology modeling techniques, the forms must be contained within a sufficient level of homology (>25–30%) [89]. Note that LBDD and SBDD are not mutually exclusive, and their combination has effectively aided several investigations requiring the screening of large libraries of compounds [90]. The CADDD process necessitates certain methodologies that are outside the scope of this paper. As a result, selecting solid reviews and perspectives to highlight technical concerns associated with various methods, while focusing on the general CADDD, is recommended. SBDD approaches are also known as implemented computer-aided approaches [91,92,93,94,95,96] (Figure 1).

### 4.1. Anticancer Small Molecule Design

There are hundreds of reports in the scientific literature where computational methods support the development of anticancer drugs [97,98]. A few examples are given here, one of which also gives an idea of how these computer-aided tools are frequently employed in anticancer drug discovery. One particularly good example is the recent development of human aromatase (HA) inhibitor. HA is responsible for transforming androgens, resulting in estrogens, and this is a well-known primary therapy for ER-positive breast cancer. HA is like a cytochrome P450 with a concealed catalytic site. In 2012, two scientists named Sgrignami and Magistrato began investigating the pathways followed by the substrate to/from the binding site, employing computational methods [99,100,101].

In one of the reported frameworks, HA was the first atomic model on a clone of the ER membrane created from 1-plamitoyl-2-oleoyl-sn-glycero-3-phosphocholine units, and this compound was utilized at random in RAMD simulations to map examples of potential pathways. The direction of an unexpected force with known strength was fixed to a specific ligand, and the order was maintained to check whether the ligand could travel a specified threshold distance clearly in a given period. This technique enables the sampling of many unbinding actions in a smaller simulation period, in this case 100. Ultimately, the relaxing routes were aggregated to identify multiple typical entry/exit channels, and then they were evaluated using the guided MD (SMD) technique. The SMD simulations, unlike RAMD simulations, apply a known-direction force to the ligand, causing it to migrate away from the binding site at a steady speed. It is required to detach the ligand, and this was employed as a test of whether the channel′s permeability was reduced due to this process.

### 4.2. Computational Method for Anticancer Peptide Design

For a long time, peptides were thought to be a niche market with limited growth prospects. This was primarily due to the failure of these molecules to cross the plasma membrane, their physiological instability, low/minimal oral absorption, and the crucial roles that the amino acid chain plays in hormonal signaling [102]. However, they have many advantages, including the potential to substitute natural agonists and the ability to target interactions between protein substances. A range of approaches, including the use of non-natural amino acids, framework alterations, and novel formulations, has helped to overcome these disadvantages, resulting in a significant increase in peptide drug production. A structure-based approach is preferred when performing computational peptide design (CPD). The primary sources of peptide sequences for therapeutic peptide design are the structures of protein–protein complexes; however, this type of knowledge is not always available, and computational chemistry may therefore contribute significantly to this. First, in conducting a computer-aided analysis to develop an amino acid chain′s affinity and specificity for a specific target, it is vital to build an accurate indicator of the peptide–target complex if possible or more bioactive chains of amino acids from random libraries or genuine sources if even a crude model is not available. Since peptides explore a larger conformational space than small compounds, consolidated docking techniques are often employed in drug development; however, these are ineffective for this type of research. Instead, where necessary, modern docking algorithms combined with experimental restrictions may aid in the selection of the proper structure [103,104]. MD simulations have estimated that this method may be utilized to assist in developing changes in peptides to improve affinity and specificity, once a model of the complex is provided [105,106].

Spodzieia et al. [107] discuss the utilization of multi-protein structures in neoplastic peptide computer-aided design. Their research aimed to develop a peptide blocker of the herpes simplex virus access regulator (HVEM) protein, which is abundantly expressed in melanoma cells and has been identified as a target for anticancer therapy. According to visual examination of the structure of the interaction of HVEM and the B and T lymphocyte attenuator (BTLA) polypeptide, removing a 17-residue amino acid from HVEM (positions 23–39) could prevent the HVEM–BTLA combination from forming. Ten-nanosecond simulations of MD supported the peptide′s ability to bind BTLA, which preliminarily corroborated this theory. Experiments revealed that the amino acid chain could effectively inhibit interactions between protein substances. Nevertheless, this impact is primarily due to the appearance of an available cysteine residue in the amino acid chain, suggesting that the observation could be due to the formation of a covalent bond between BTLA and the protein rather than a compound with the same framework as seen in the X-ray studies.

## 5. Structure-Based Approach

The sequencing of the human genome has created a paradigm shift in drug discovery techniques on a large scale as a result of structure-based drug design. It allows for a better understanding of many types of cancer-associated developments and the detection of cancer targets (SBDD). SBDD must be employed to identify anti-neoplastic agents with a variety of structures by studying binding site interactions and specificity factors using cutting-edge technology such as the 3D architectures of proteins in cancer that are relevant for treatment. Two types of structure-based techniques have been identified: protein-ligand-based complexes and protein-based strategies. Regarding SBDD, studies on the target structural information in complex ligands are helpful in this drug discovery method. The principal interaction between the target protein and ligand is extracted from the protein-ligand-based complex, giving an excess of information on either the activity or inhibitory actions. A protein-based technique can be employed if a protein-ligand-based strategy is not available, where attributes of the relevant protein binding data can be transformed into pharmacophore properties. Studies on contemporary drug development leverage one or two of the SB approaches outlined below if a cancer target’s structural information is accessible [108].

### 5.1. Docking of Molecules

Molecular docking is a standard structure-based methodology used in logical drug design to examine and predict sequences and interaction relations between ligands and receptor proteins [108]. Depending on how adaptable the ligands utilized in the computational method are, molecular docking is characterized as rigid docking or flexible docking [109,110]. The inflexible docking approach, also known as a critical approach, focuses solely on the fixed geometry and structural and chemical reciprocity between the ligands and targeted proteins, ignoring elasticity and the induced-fit hypothesis [111]. Rapid and highly productive rigid docking is utilized extensively in drug discovery, with many small molecular databases, and it is time-efficient. However, in a flexible docking approach, this information would be more precise and complex. Different types of software are available for docking, such as Glide, FlexX, DOCK, AutoDock, Discovery Studio, Sybyl, and so on.

There are three basic phases in the molecular docking process. It is first necessary to prepare the small molecule and target protein structures. The ability to anticipate conformations, orientations, and positional spaces in the ligand binding site is its second use. Using the techniques of systematic and stochastic searching, conformational search algorithms complete this goal of predicting the conformations of binary compounds. There are three types of systematic search methods: exhaustive search, fragmentation, and conformational ensemble. Stochastic approaches, on the other hand, include: (i) Monte Carlo (MC) methods, (ii) tabu search methods, (iii) evolutionary algorithms (EA), and (iv) swarm optimization (SO) methods. Finally, these algorithms assess the potential binding free energy, which works in conjunction with the scoring function to identify the molecules that have a higher propensity for binding to targets during molecular docking. There are four primary categories of scoring functions: I functions for consensus scoring, empirical scoring functions, knowledge-based scoring functions, and force-field-based scoring functions, among other options [88,112].

### 5.2. Pharmacophore Mapping Based on Structure

Over time, there has been a significant change in one of the most useful technologies, i.e., pharmacophore mapping. This has been considered during the drug development process in recent decades. Various structure-based techniques have been used for pharmacophore modeling. Virtual screening, de novo design, and lead maximization have all benefited from pharmacophore modeling [113,114]. In relation to the availability of ligand structures, there are two different types of SBP modeling methods: target-binding site-based and target-ligand-based approaches [115]. The target-ligand complex method makes locating the protein′s ligand-binding pocket and determining the important ligand–protein interactions uncomplicated. Ligandscout [116], pocket v.2 [117], and GBPM [118] provide examples. It is worth noting that where ligands are unknown, they cannot be used. The macromolecule employed in Discovery Studio [119] without a ligand-based technique is an actual example that is not dependent on ligands or receptor–ligand associations. LUDI [120] is software that characterizes the interactions within the binding site as pharmacological properties. While this strict SBP technique has benefits in specifying a binding pocket′s total interaction potential, the generated interaction maps frequently have multiple unprioritized interactive features.

## 6. Drug Development Based on Ligands

### 6.1. Searching for Similarities

The concept of molecular similarity, which is the basis and motivation for ligand-based techniques in drug development, lies behind these techniques. Due to their structural similarities, compounds based on this concept have a tendency to have identical physiological activities [121]. The structural information of the active ligand that interacts with the target protein can be utilized as a query template to identify and predict other chemical entities with a related property. On the other hand, there is a ligand-based drug discovery technique that is based on this structural data. This method is referred to as an indirect protocol for pharmaceutical research, since it only requires the structure of known active small molecules. This provides a choice when a protein′s 3D defined structure is unknown or cannot be predicted. In order to enhance medication pharmacokinetics, including ADMET properties, this technique is frequently employed in silico to screen novel ligands with fascinating biological activities and to maximize ligand biological activities (adsorption, distribution, metabolism, excretion, and toxicity). This fundamental method is applied worldwide and is based on molecular descriptors.

### 6.2. Ligand-Based Pharmacophore Mapping

Ligand-based pharmacophore modeling is a more specific method of improving pharmacophore models based on a collection of active chemicals similar to molecular descriptors. According to the IUPAC, a pharmacophore is “a set of spatial and electrical features required to achieve maximum supramolecular associations with predefined biological active targets and to initiate biological reactions” [122]. In this method, the structural overlap of major molecular properties obtained from potentially active compounds or in the specific binding site space is employed as a way to depict the most likely chemical features. Here, very newly discovered compounds can be matched and can exhibit a wide response with the enhanced pharmacophore, enhancing effectiveness against the specific target protein. They can then be used as candidates for further investigations. As a result, adhering to this critical computational strategy method aids in promoting and guiding drug discovery without using macromolecular structures [123]. Ligand-based pharmacophore modeling provides a better training set of drugs that have the same receptor.

### 6.3. Modeling with QSAR

Another ligand-based technique known as QSAR (quantitative structure–activity relationship) involves assessing the bioactivities of pharmaceuticals using a variety of molecular descriptors (MDs) or fingerprints (FPs). The function of QSAR modeling is to describe the activity′s response to the target based on the ligand characteristics. Support vector machine (SVM), random forest (RF), polynomial regression (PR), multiple linear regression (MLR), and artificial neural networks (ANNs) are some of the ML and dynamic programming (DP) methodologies utilized to create QSAR models [124]. Unlike in pharmacophore models, the biological actions of QSAR can be quantified. Further molecular design applications are accessible, such as developing new molecules, optimizing lead compounds, and predicting new structural lead compounds in drug discovery. There are also current advancements in science such as 2D-QSAR and the sophisticated 3D-QSAR that can be used in computational methods.

## 7. Artificial Intelligence Aids in the Discovery of Anticancer Drugs

Promoting the development of multiple new anticancer medications through the use of computational drug design has become a watershed moment in this field. Authorized medications identified using a computational technique include Gefitinib [125], Erlotinib [126], Sorafenib [127], Lapatinib [128], Abiraterone [129], and Crizotinib [130]. Anticancer drug research has progressed slowly but steadily through the use of computational approaches. SR13668, for example, was developed from indole-3-carbinol utilizing the PH4 layout. Rodrigues et al. recently succeeded in identifying a very effective inhibitor for 5-LOX (lipoxygenase) using a computer-learning-based technique based on physicochemical and pharmacophore properties [131,132]. The introduction of AI has led to a significant evolution of the in silico design of anticancer medications; state-of-the-art learning algorithms can help develop the good chemical characteristics required for novel compounds [133]. Jann et al. used variational autoencoders to create the first ML-based antineoplastic chemical synthesizer, proving that compound synthesis may be selective against compounds with high expected resistance to a particular cancer [134]. They incorporated the disease′s bimolecular characteristics into lead chemical identification efforts. This technique could change the development of anticancer medication in silico. Artificial intelligence research and the underlying machine learning algorithms may be especially useful for finding successful pharmaceutical treatments for complex disorders. A prime example of this is cancer, which is one of the top causes of death in the world. Most of the aggressive subtypes of cancer have not yet been successfully treated using a systematic scientific method. Cancer develops when abnormal cells proliferate uncontrollably in one area of the body and can invade and harm nearby healthy tissue and organs. It is a very complicated illness with more than 200 subtypes, each of which requires a unique diagnosis and course of treatment. Scientists have developed sophisticated profiling methods to measure these aberrations and utilize them to personalize medicines, since cancer develops from aberrations in the genomic materials of the cells. The most popular cancer therapies are still radiotherapy and chemotherapy, which use high-energy X-rays to kill most of the proliferating cells. Even though these treatments have the potential to be extremely hazardous and are not designed to target the particular set of genomic abnormalities that give each tumor its own individuality, they can occasionally be effective in decreasing or eliminating malignant cells. Researchers could benefit from artificial intelligence techniques by analyzing the intricate genomic makeup of each individual tumor, to create precise predictions of therapy response. As a result, better treatments for specific patients could be found, which would be a significant step toward personalized medicine [88,135,136].

## 8. Discovering New Drug Binding Sites through the Use of MD Simulation

Knowledge of multiple protein–ligand interrelationships is vital to some critical biological processes. Understanding the work of endogenous ligands and synthetic drug molecules requires the identification and characterization of LBP. GPCR is a target often used in the development of new drugs [137]. A recent study revealed that ligands bind to various allosteric sites other than the intended binding sites, in addition to orthosteric points [138,139,140]. Primary computational methods for predicting functional areas such as 3D ligand sites and others were discussed in a recent overview. However, these reporting tools frequently generate many potential ligand binding sites, making it difficult for the user to determine which active pocket of the structure is correct for chemical or drug binding. In recent years, approaches based on molecular dynamics (MD) have been used to circumvent this limitation. Supervised MD, for example, is an effective method for precision sampling and ligand-binding-site identification [141,142,143]. The MD simulations revealed an extra sodium ion in the region of the orthosteric binding site [144], and could be used to recognize allosteric sites in protein kinases, Ras proteins, and *Staphylococcus aureus* Sortase, among other things [145,146,147].

## 9. Integration of Structure- and Ligand-Based Approaches

Numerous studies have pointed out that structure-based techniques and ligand-based techniques are independently viable methodologies used in the identification of anticancer medicines [148,149,150,151]. Selecting certain structure-based and ligand-based procedures and using them in combination to identify active chemicals is the most typical strategy for combining the approaches. In vitro mTOR kinase assays validated active compounds with low Tanimoto similarities, indicating that the integrated VS approach may swiftly find structurally distinct inhibitors for a specific target [152].

EGFR LB models for the VS of the Molecules database were generated using ligand-based Laplacian-modified NB classifiers (approximately 6M compounds). To isolate dual EGFR-BRD4 leads, predetermined EGFR hits were docked into an ensemble of BRD4 protein form. Experiments were conducted on several compounds, revealing one lead candidate with adequate dual action that could be further improved [153]. Despite the increasing availability of structural data, several therapeutically significant protein families still lack structures for computational drug development. The fundamental restriction in employing an integrated strategy is deciding which methods to integrate and how many to incorporate to generate superior and non-redundant output [154].

### 9.1. Pseudoreceptor Modeling

Pseudoreceptor models can associate SBDD and LBDD techniques by depending on surrogate three-dimensional receptor structures that modify the contour and volume of the binding region and the critical interaction characteristics between the receptor and ligand. In order to verify the correct direction of the receptor–ligand interaction properties integrated into the models, the bioactive conformation of these compounds should be determined using experimental methods such as mutation studies. Partition-based, grid-based, peptide-based, isosurface-based, atom-based, and fragment-based techniques are some of the categories covered in Tanrikulu′s review [155,156]. Pseudoreceptor modeling has been used in much cancer-related computational research. For example, Rödl et al. used molecules discovered through similarity search algorithms as reference structures to create a 5-Lipoxygenase (5-LO) pseudoreceptor model. The VS experiment revealed potent and non-cytotoxic inhibitors that could be employed as a source of novel scaffolds for lead optimization. This supplied the interaction pattern required for binding [157,158,159,160]. It is also interesting to note that pseudoreceptors cannot wholly mimic the actual size and shape of the expected receptor’s binding pocket. Therefore, this framework, based on a set of reference molecules, might favor compounds with identical structures [11].

### 9.2. Proteochemometric Modeling

Lapinsh et al. established the term “proteochemometrics” when they developed a new methodology for examining receptor–ligand interaction data in their work. They investigated the binding information of chimeric receptors and their ligands [161]. This skill can be used simultaneously to represent the interactions of a group of molecular entities with a group of receptors, as opposed to explaining the important contacts for an individual ligand and an individual receptor. As a result, this technique may be utilized to deduce the relationships between a group of linked QSAR datasets. Furthermore, the PCM model developed can be applied to newly identified targets connected to the series under investigation [162]. Wu and colleagues explored the use of PCM models to screen for selective HDAC inhibitors, and their work is an excellent illustration of the use of PCM modeling in the hunt for anticancer drugs. The best model used in this study accurately predicted inhibitory actions for each HDAC inhibitor, and it was able to determine the drug’s class- and isoform-selectivity, leading to the discovery of leads with less negative consequences [163]. COX-2, which has been linked to colorectal cancer and whose suppression is a promising technique for the development of anticancer treatments, is a more recent example [164,165,166].

## 10. Current Trends in Computational Approaches for Anticancer Drug Delivery Systems

Drug inventory research mainly focuses on the “one drug, one target” concept, in which unique chemical entities are discovered and created with a specific, well-defined target. As a result, the core concept of “one drug, multiple targets” has manifested as a universal concept in drug development, including poly-pharmacology and drug repositioning (DR) methodologies in cancer studies. Thus, poly-pharmacology is gaining popularity as the next anticancer drug discovery paradigm. The interaction of drugs with several targets, which may interfere with a single or multiple disease processes, is the fundamental idea of poly-pharmacology [167,168]. Data mining, chemogenomic methodologies (integrated LBSB methodologies and structure-based or ligand-based methodologies), and network pharmacology are promising computational techniques in this field, including machine learning methods, drug–target interactions, and drug side-effect profiles for in silico computational profiling and DR [168,169]. Within the Hsp90 interactome, an in silico poly-pharmacology approach was recently developed for choosing interesting target combinations and creating targeted chemical libraries [170]. Drug repositioning or drug repurposing is the process of finding new uses for already approved treatments, particularly generic pharmaceuticals. It is thought to be an excellent technique for drug discovery because it manages risks and is cost-effective. Antihistamines such as astemizole have been withdrawn from the market due to their side effects, as they cause arrhythmias at higher doses.

The same drug was repurposed as an anticancer drug that slowed tumor growth [171]. Another study used an integrated in silico DR technique to find DR candidate anticancer medicines for lung cancer, glioblastoma, and breast cancer using the expression signature and target signatures acquired from the LINCS chemical structure [172]. In a recent work, Huang et al. developed a new DR pipeline that utilized topological graph theory and machine learning techniques to examine four biological processes that are enriched for lung cancer, with potential therapeutic drugs, microarray datasets, and targeted genes against NSCLC [173]. Another study described a novel systems pharmacology strategy for repurposing metformin as a precision cancer treatment [174]. It has been suggested that in the post-chemical genomic era, the DR field will extend to include large-scale omics impacts such as those of proteome, transcriptome, and metabolome [172].

## 11. Successful Stories in Computational Drug Discovery

**Figure 2:**
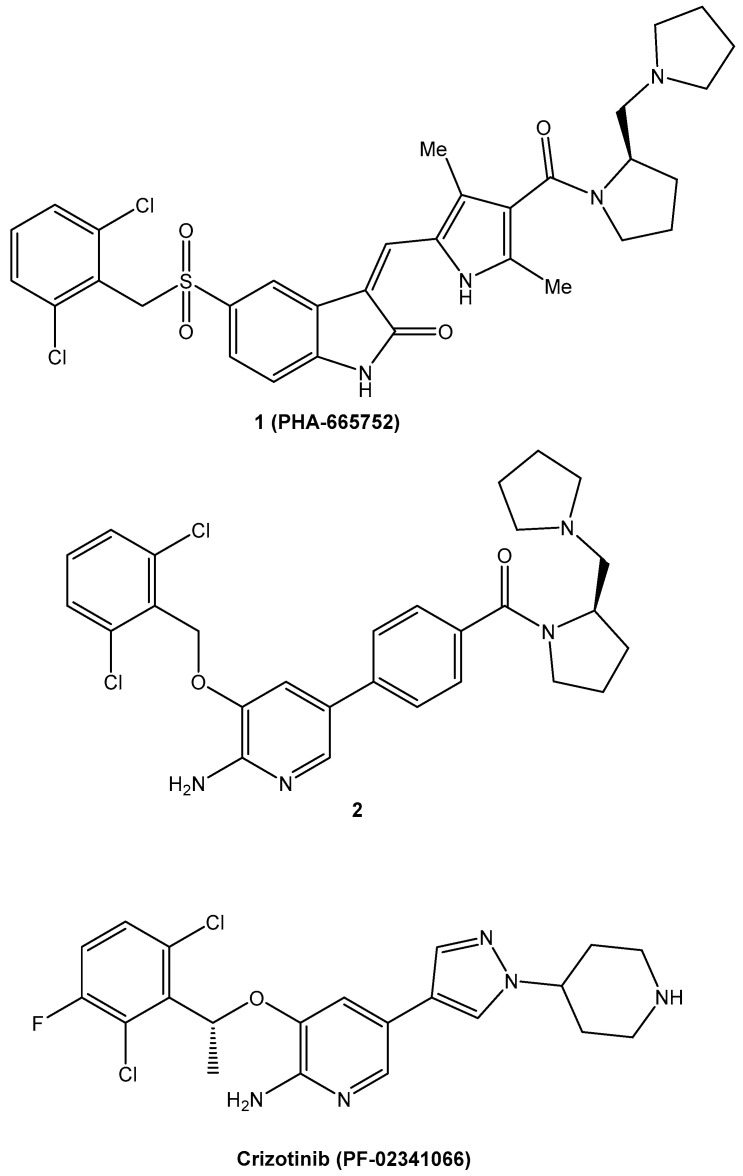
Successful uses of computational tools in the search for anticancer drugs.

Modern drug discovery benefits from computational methods. Almost every step of the drug discovery pipeline uses computational methods. Properties, time required, and anti-neoplastic drug functioning have demonstrated improvements through computational methods. Computational methods are regarded as a potent success of anti-neoplastic drug development (Table 4). Some benefits of this computational method for small drug molecules have been discussed. This type of drug is used in cancer treatment or in clinical trials. Crizotinib′s development is an important example of the use of structure-based design methodologies [175,176]. Crizotinib, which was accepted by the FDA in 2011, is a specific and powerful cMet/ALK dual antagonist [177]. c-Met, also termed HGFR, and its endogenous ligand HGF (hepatocyte growth factor), are important regulators of a variety of cell functions [178]. Increased spreading of the c-Met polypeptide has been found in a variety of human malignancies (including SCLC and NSCLC) [177], as has aberrant c-Met signaling function in a variety of solid and blood tumors. As a result, c-MET is a compelling prospective cancer target. The study began with the evaluation of a sequence of 3-substituted indolin-2-one analogues for c-MET suppression, representing a powerful family of kinase inhibitors. Compound **1** (PHA-665752, Figure 2), among the analogues, demonstrated substantial efficacy in vitro and in vivo against the c-MET autophosphorylation method and the resulting physiologic authorizations. Compound **1** (PHA665752)′s poor drug-like qualities, on the other hand, hampered its future investigation. The major blocker interaction site was identified by combined crystalline structure study of compound **1** with the kinase domain of c-MET, allowing for better drug design. In conjunction with redrawing the intermediate rings of compound **1**, a novel 5-substituted 3-benzyloxy-2-aminopyridine family was developed (PHA-665752). Compound **2** showed potential suppression of c-MET between these recently developed compounds. It should be mentioned that lipophilic effectiveness (LipE) was used as a metric for determining efficacy, to track the maximization process. A docked shape of compound **2** with the c-Met kinase domain was used to assist the use of framework design strategies to further optimize the c-Met suppressive strength. Crizotinib (PF-02341066), with excellent restriction of tumor growth and strong drug effectiveness, was developed after improvement of the 3-benzyloxy group (the functional group at the 5-position of the 2-aminopyridine) and evaluation of the chiral point. Furthermore, Crizotinib has proven to have excellent therapeutic effects against lung cancer, lymphoma, and esophageal malignancies by inhibiting c-MET gene proliferation (Figure 2) [179,180].

**Figure 3:**
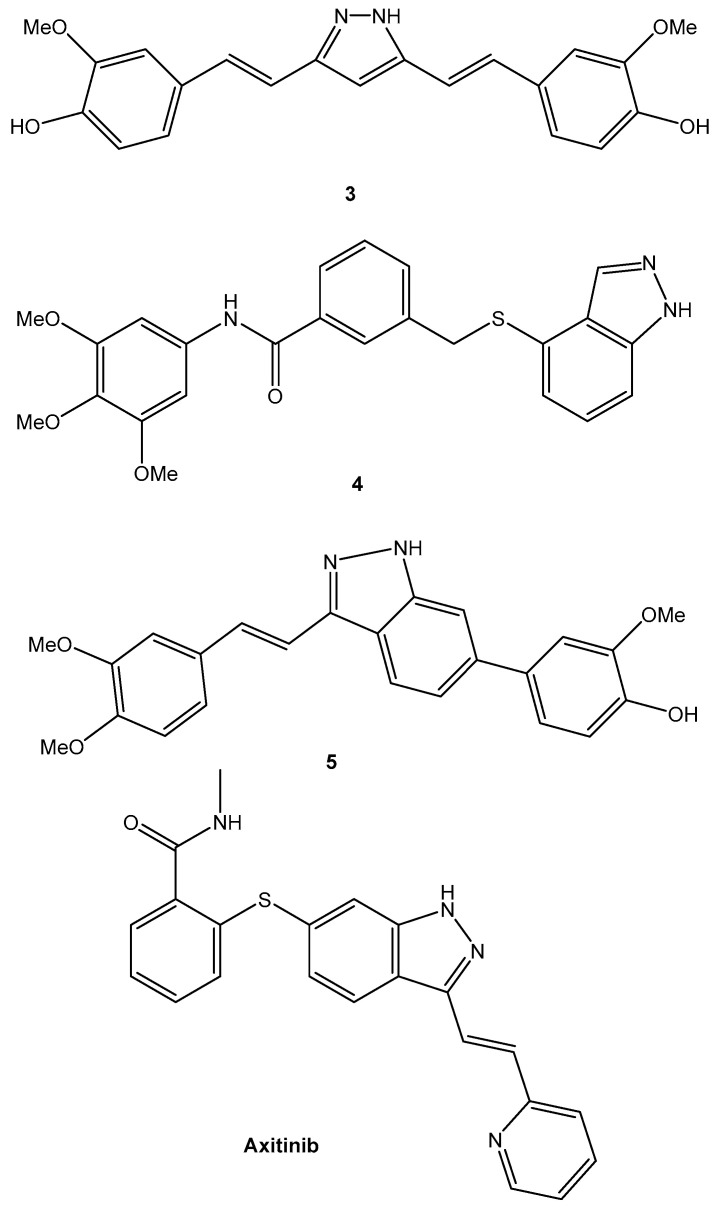
Successful applications of computational methods in anticancer drug discovery.

The FDA authorized axitinib (AG-013736) in 2012 as a novel therapeutic for advanced renal cell carcinoma to treat VEHG. The VEGF kinase domain in the DFG-out conformation is where axitinib binds to act as an inhibitor. Axitinib was created using a structure-based drug design approach. Vascular endothelial growth factor (VEGF) family members serve as significant signaling network regulators that are involved in angiogenesis. Tumor cells have been found to express VEGF signaling, which is essential for the development of malignant illnesses. VEGFRs function as ligands in the VEGF signaling network, as they are the main VEGF receptors. The tyrosine kinases (RTKs) known to be VEGF receptors include VEGFR-1 (also known as FLT1), VEGFR-2 (also known as FLK1 and KDR), and VEGFR-3 (also called FLT4). The pan-kinase inhibitor’s ability to block the activity of VEGFRs against VEGFR-1, VEGFR-2, and VEGFR-3 has been demonstrated to offer an effective method of developing anti-angiogenic drugs. All of these derivative compounds were shown to have strong inhibitory effects (Figure 3). The cellular potency and the favorable physicochemical and PK characteristics of axitinib were significantly improved. Axitinib and pembrolizumab were recently approved as first-line anticancer treatments for renal cell carcinoma.

## 12. Conclusions and Future Perspectives

Cancer is a genuine threat to individuals′ well-being. About 9.6 million individuals are affected each year by this disease. Malignancies have exceeded coronary illnesses as the primary source of mortality in people. Progressing up-to-date anticancer therapies takes twelve years and costs approximately USD 2.7 billion. The discovery of newer potent drugs for treating cancer has been challenging, partly due to the limited knowledge we possess on the in-depth mechanisms of each type of cancer. Development of a new drug is expensive and time-consuming. Computational techniques might be helpful in the drug discovery process with a large range of applications, for example, protein-association network examination, drug target forecasting, restricting site expectation, virtual screening, and numerous others. These approaches may help to accelerate the development of newer, more effective drugs for cancer and malignancies. More advanced techniques such as retro-manufactured routine arrangement, drug framework age, and medication restricting fondness expectations have been gaining much significance lately with the advent of AI. The applications of computational models along with newer advanced technologies may help accelerate the development of effective drugs against malignancies. We outlined the methods for identifying anticancer drugs in the sections above. We primarily covered four topics in our discussion of these techniques′ applications: precision cancer therapy, drug positioning, and drug design forecasting for medication combinations. There is no denying that the generation of an enormous amount of multi-omics data offers excellent possibilities for precise and effective anticancer medication discovery at an affordable cost. Despite the significant progress made to date in improving the effectiveness of predictive methods, there are still many difficulties in the real world. Firstly, many computer studies that have already been performed incorporate pre-existing knowledge such as PPI networks and biological pathways. The integrality and accuracy of computational predictions are, however, severely constrained by the preceding data′s continued high sparsity. Additionally, more investigation into context-specific therapies for drug response prediction is required. Since there are at present only a few datasets available for particular tissues or medications, prediction models have mostly been based on pan-cancer data studies, which do not take context-specificity into account. One of the key criteria for future projections for certain types of cancer is the differences in the molecular profiles of the cancer cell lines between tumor types. Additionally, the majority of the current predictive approaches for drug development rely on the transcriptome profiles of cancer cell lines, which are molecular profiles of cancer cell lines. Cancer cell lines, it has been demonstrated, do not accurately replicate the molecular abnormalities seen in patients. To achieve therapeutically relevant research, bioinformaticians should be well-versed in the limits of cell lines. The models are also less extendable and useful in clinical contexts when using methods that rely on sparser data types, such as CMap-based models, for which data are only accessible for a small number of cell lines in a limited range of tissue types. The primary causes of the failure of computational drug development are the selection of cell lines that inadequately reflect the tumor biology and the lack of appropriate cell lines for modeling response for certain malignancies. The following techniques may be used to potentially overcome the aforementioned constraints of computational approaches to drug discovery. Firstly, the applicability of computational prediction models in clinical practice would be improved by using data types that are less dependent on the data types themselves and more similar to patients in an in vitro scenario. Secondly, it may be possible to create more precise predictive models by combining various independent datasets. Thirdly, model validations using patient data and clinically applicable animal models are still required. The integration of clinical data may be more effective for forecasting cancer drugs for therapeutic purposes, which may be accomplished through collaborations with doctors. This is the final and most crucial step in the translation to the clinical process.

## Figures and Tables

**Table 1:** Sources of data for determining the correlations between cancer and genes.

Database	Simple Explanation	Reference
Gene Expression Omnibus (GEO)	GEO is a free, open-access repository for functional genomics data that accepts submissions of MIAME-compliant data.	Gene expression omnibus. Available online: http://www.ncbi.nlm.nih.gov/geo (accessed on 23 January 2022)
The Cancer Genome Atlas (TCGA)	Genomic statistics from >10,000 patient tissue samples from >30 prevalent cancers, such as exome, SNP, methylation, mRNA, miRNA, and clinical.	The Cancer Genome Atlas Program. Available online: http://cancergenome.nih.gov (accessed on 23 January 2022)
Genetic Association Database (GAD)	A database of information on genetic associations with serious illnesses and disorders.	Gender and Development Program. GAD Activities Sex Disaggregated Data. Available online: http://www.tapi.dost.gov.ph/resources/gad-databases (accessed on 23 January 2022)
Catalogue Of Somatic Mutations In Cancer (COSMIC)	A thorough resource for learning about somatic mutations’ effects on human cancer.	Catalogue Of Somatic Mutations In Cancer. Available online: https://cancer.sanger.ac.uk/cosmic (accessed on 23 January 2022)
Online Mendelian Inheritance in Man (OMIM)	Relationship between genetic traits, especially diseases, and genes.	An Online Catalog of Human Genes and Genetic Disorders. Available online: http://www.omim.org (accessed on 23 January 2022)

**Table 2:** Data sources for determining the correlations between drugs and genes.

Database	Simple Explanation	Reference
Therapeutic Target (TTD)	A database that offers details on the diseases targeted, the investigated and undiscovered therapeutic protein and nucleic acid targets, the relevant methods, and the medications that are specific to each target.	Therapeutic Target Database. Available online: https://openebench.bsc.es/tool/ttd (accessed on 23 January 2022)
Genomics of Drug Sensitivity in Cancer (GDSC)	A database of 138 identified anticancer compounds (on average 525 cell lines studied for each drug) representing more than 1000 distinct cancer cell lines.	Genomics of Drug Sensitivity in Cancer. Available online: http://www.cancerrxgene.org/download (accessed on 23 January 2022)
DrugBank	Complete drug target data, including information on sequencing, structure, and route, together with detailed drug (i.e., chemical, pharmacological, and pharmaceutical) data.	Drug bank online. Available online: http://www.drugbank.ca/ (accessed on 23 January 2022)
PharmGKB	A freely accessible online knowledge repository that collects, organizes, synthesizes, and disseminates information about the influence of genetic variation on pharmacological response.	Online Knowledge Repository. Available online: https://www.pharmgkb.org/ (accessed on 23 January 2022)
Cancer Cell Line Encyclopedia (CCLE)	Genomic data, including information on DNA copy number, mRNA expression, and mutations, from more than 1000 cancer cell lines.	Cancer Cell Line Encyclopedia. Available online: https://portals.broadinstitute.org/ccle (accessed on 23 January 2022)

**Table 4:** The list of FDA-approved anticancer drugs from the National Cancer Institute database.

Name	Molecular Formula	ATC Code	Therapeutic Area	Target and Function	Year of Approval
Alpelisib	C_19_H_22_F_3_N_5_O_2_S	L01EM03	Breast cancer	PI3K inhibitor	2019 [181]
Cladribine	C_10_H_12_ClN_5_O_3_	L04AA40	Hairy cell leukemia	Adenosine deaminase inhibitor	2019 [182]
Darolutamide	C_19_H_19_ClN_6_O_2_	L02BB06	Prostate cancer	Androgen receptor inhibitor	2019 [183]
Entrectinib	C_31_H_34_F_2_N_6_O_2_	L01EX14	Non-small-cell lung cancer and solid tumors	Tyrosine kinase inhibitor	2019 [88]
Erdafitinib	C_25_H_30_N_6_O_2_	L01EN01	Urothelial carcinoma	FGFR tyrosine inhibitor	2019 [184]
Fedratinib Hydrochloride	C_27_H_36_N_6_O_3_S	L01EJ02	Myelofibrosis	Tyrosine kinase inhibitor	2019 [185]
Selinexor	C_17_H_11_F_6_N_7_O	L01XX66	Multiple myeloma	Nuclear export inhibitor	2019 [186]
Zanubrutinib	C_27_H_29_N_5_O_3_	L01EL03	Mantle cell lymphoma	Bruton′s tyrosine kinase inhibitor	2019 [187]
Abemaciclib	C_27_H_32_F_2_N_8_	L01EF03	Breast cancer	Cyclin-dependent kinase inhibitor	2018 [188]
Apalutamide	C_21_H_15_F_4_N_5_O_2_S	L02BB05	Prostate cancer	Androgen receptor inhibitor	2018 [189]
Binimetinib	C_17_H_15_BrF_2_N_4_O_3_	L01EE03	Melanoma	MEk1 and MEK2 inhibitor	2018 [190]
Dacomitinib	C_24_H_27_ClFN_5_O_3_	L01EB07	Non-small-cell lung cancer	Oral kinase inhibitor	2018 [191]
Duvelisib	C_22_H_17_ClN_6_O	L01EM04	Chronic lymphocytic leukemia (CLL) and follicular lymphoma (FL)	PI3K kinase inhibitor	2018 [192]
Encorafenib	C_22_H_27_Cl_1_F_1_N_7_O_4_S_1_	L01EC03	Colorectal cancer and melanoma	BRAF kinase inhibitor	2018 [190]
Gilteritinib Fumarate	C_62_H_92_N_16_O_10_	L01EX13	Acute myeloid leukemia	Tyrosine kinase inhibitor	2018 [193]
